# Inherent structure length in metallic glasses: simplicity behind complexity

**DOI:** 10.1038/srep12137

**Published:** 2015-08-06

**Authors:** Yuan Wu, Hui Wang, Yongqiang Cheng, Xiongjun Liu, Xidong Hui, Taigang Nieh, Yandong Wang, Zhaoping Lu

**Affiliations:** 1 State Key Laboratory for Advanced Metals and Materials, University of Science and Technology Beijing, Beijing, 100083, China; 2 Chemical and Engineering Materials Division, Oak Ridge National Laboratory, Oak Ridge, Tennessee 37831, USA; 3 Materials Science and Engineering Department, University of Tennessee Knoxville, Knoxville, Tennessee 37919, USA

## Abstract

One of the central themes in materials science is the structure-property relationship. In conventional crystalline metals, their mechanical behaviour is often dictated by well-defined structural defects such as dislocations, impurities, and twins. However, the structure-property relationship in amorphous alloys is far from being understood, due to great difficulties in characterizing and describing the disordered atomic-level structure. Herein, we report a universal, yet simple, correlation between the macroscopic mechanical properties (i.e., yield strength and shear modulus) and a unique characteristic structural length in metallic glasses (MGs). Our analysis indicates that this characteristic length can incorporate effects of both the inter-atomic distance and valence electron density in MGs, and result in the observed universal correlation. The current findings shed lights on the basic understanding of mechanical properties of MGs from their disordered atomic structures.

Modulus and strength of metallic materials are key properties for their engineering applications^1^,^2^, and the knowledge of structural information is prerequisite for understanding the deformation behaviour^3^,^4^. In crystalline materials with long-range periodic atomic packing, the relationship between their structure and mechanical properties has been theoretically developed and relatively understood^5^,^6^,^7^,^8^. However, in glassy materials, especially metallic glasses (MGs), such a structure-property correlation is still missing due to the difficulties in decoding the atomic packing in these disordered solids. Several efforts have been attempted to reveal a scaling behaviour between the glass transition temperature and strength^9^,^10^,^11^,^12^,^13^, and the strength has also been found to be proportional to the elastic modulus, corresponding to a nearly-constant yield strain^11^,^12^,^13^. However, a direct correlation between the characteristic length in the “structure” and these mechanical properties of MGs has not yet been established.

For amorphous solids, the total structure factor, S (q), and the resolved pair distribution function (PDF), g(r), are particularly useful in characterizing the non-crystalline structure (q is the wave vector in reciprocal space and r is the nearest neighbour distance between two atoms). From these statistical data, the atomic distribution within a given volume can be well described^14^,^15^,^16^,^17^,^18^. In principle, S (q) and g (r) was related by Fourier transformation^19^, and the first peak position of S (q) plots, usually denoted as q_1_, specifies the wavelength (λ = 2π/q_1_) of g (r) at the medium-range length scale or above^20^,^21^. Particularly, q_1_ was found to be a key structural quantity to reflect critical characteristics in atomic network of amorphous matters, such as intermolecular bonding in amorphous ice, interconnected layers of some borates, and the weak chain in chalcogenide glasses^22^,^23^,^24^. It was found that different metallic glasses may have different q_1_ values and thereby different mechanical properties. For example, Zr-based BMGs generally have a smaller q_1_ and lower yield strength, as compared with Fe-based BMGs (a typical example is shown in Fig. 1). To find out whether there exists a relationship between q_1_ and mechanical properties of MGs, most of available data in literature regarding the q_1_ values and elastic properties of MGs was complied in Table 1^25^^–^92. By analyzing the structural length and mechanical properties of these MG alloy systems, we discovered a universal relationship between the elastic properties (i.e., the yield strength and moduli) and the wavelength λ (i.e., 2π/q_1_).

Figure 2a indicates a strong dependence of the yield strength on the specific length scale λ within the experimental error of measurements, and the fitted scaling relationship is:


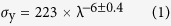


Where σ_y_ is the yield strength (in unit of GPa) and λ is the wavelength (in unit of Å). Note that the above relationship is generally applicable to different types of MGs, independent of their chemical compositions. With increasing λ, the yield strength of MGs rapidly decreases. For example, Co- and Fe-based MGs containing metalloid constituents have relatively small λ, but high yield strength, whereas Ca- and RE- (rare earth) based MGs exhibit large λ but low yield strength.

Elastic constant, especially the shear modulus, has been shown to be the dominant parameter controlling atomic shear and, consequently, the yielding of metallic glasses^10^,^11^,^12^,^13^. Using the current data, an intriguing correlation between the shear modulus, *G*, and the specific length scale λ is also established as shown in Fig. 2b:


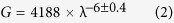


where G is the shear modulus (in GPa). Similar to the yield strength, the shear modulus is also strongly dependent upon the specific length scale, i.e., the wavelength λ, with a similar power index. This observation further suggests that yield strength and shear modulus of MGs, in fact, may have the same atomic structural origins^11^,^12^,^13^. Combine Eqs. (1 and 2), we have the shear strain limit of MGs, 
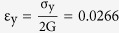
, which is in excellent agreement with the value of 0.0267 obtained by Johnson and Samwer^11^, further validates the above correlations. Noted that systematic errors associated with the experimental measurements of the yield strength and the diffraction patterns collected from different groups might have contributed to the data scattering shown in Fig. 2.

As shown above, the elastic properties (elastic moduli and yield strength) can be directly assessed by the simple parameter λ, in despite of the complex chemistry. In structural materials, the forces that affect elastic stiffness are predominantly electrodynamic, that is, a combination of electrostatics and dynamic exchange forces associated with various quasi-particles, including phonons, photons, electrons, and protons^93^,^94^. For metallic materials, the force usually comes from electrostatic interactions resulted from the distribution of electron charge, and the elastic modulus is determined primarily by atomic spacing and valence electron density^93^,^94^. It is, therefore, pertinent to establish a correlation between the λ value and the atomic spacing and valence charge in BMGs.

It is necessary to point out that the physical meaning of q_1_ or λ (2π/q_1_) has been extensively studied in amorphous matters, for example, inorganic glasses bonded by directionally covalent bonding. In such materials, it was proposed that 2π/q_1_ could be interpreted as spatial repeat distance of structural unit^15^. For MGs, which exhibit long-range disordered packing with atoms bonded by nondirectional metallic bonding, the physical significance of q_1_ has also been discussed in recent years^12^,^14^,^19^. As mentioned before, q_1_ identifies the wavelength (λ = 2π/q_1_) of the correlation function g(r) above the first nearest neighbour (i.e., the portion at the high r region of the PDF curves). In terms of atomic packing, this wavelength actually quantifies the distance between the two adjacent atomic neighbors and therefore represents the average inter-atomic distance in MGs^14^,^95^.

Based on the spherical-periodic theory, q_1_ (i.e., 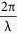) is essentially the Fermi-sphere diameter 2k_F_ for MGs, and λ is the Friedel wavelength^96^,^97^,^98^. The spherical Friedel oscillations in the effective pair potential due to the electronic interaction are similar to those in the structural PDF curves, namely, the electronic wave vector 2k_F_ equals to the structural wave vector q_1_ (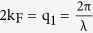)^97^,^98^,^99^. In other words, when the Fermi surfaces of the electrons coincide with the quasi-Brillouin boundaries, a pseudo gap is expected occur in the energy bands for MGs and a corresponding minimal of density of states at the Fermi level, which makes MGs in a relative stable state^98^,^100^. Therefore, q_1_ (or λ) can be treated as an indicator of the electronic interaction in MGs. As a matter of fact, the universal binding-energy relation with interatomic separation has been found in a wide range of crystalline materials^101^,^102^, which was believed to be resulted from the scaling between electron density parameter and interatomic distances^101^,^102^. However, a universal relation between elastic modulus which is the second derivative of binding energy and interatomic distances was not observed due to crystallographic lattice anisotropy. In MGs, as discussed earlier, q_1_ (or λ) can represent relative electron density, indicating the proposed universal binding-energy relation also holds well. Due to the atomic isotropic disordering structure of MGs, the universal correlation between the elastic modulus and interatomic distance can also be derived.

It should be noted that there is no distinct relationship between the yield strength and the nearest neighbour distance (r_1_), as shown in Fig. 3a. The data points are much more scattered, as compared with those in Fig. 2a. For example, even at the same r_1_ value of ~2.6 angstrom, a large scattering in the yield strength from 1 to 5 GPa was observed. From the atomic packing point of view, r_1_ represents the first nearest neighbour which can be more easily affected by local atomic environment. Although λ is at the scale of 2-3 angstrom, nevertheless, it actually specifies the wavelength (λ=2π/q_1_) of g(r) at the large r range (as shown in Fig. 3b)^20^,^21^ and reflects the average atomic distance of MGs. Therefore, it can be seen that the average packing characteristic is more determinative to the global elastic properties, rather than the nearest neighbour distance or the localized atomic packing features which may be more contributable to the plastic deformation behavior.

It has been long recognized that theoretical strength and modulus of a material depend on their bonding nature, and specifically the bulk modulus is the second derivative of the binding energy with respect to the interatomic distance^1^,^3^,^4^. For instance, molecular crystals are bonded by dipole-dipole forces (similar to that in solid inert gases) and their bulk moduli scale with inter-particle distances raised to the -3 power^94^. For metallic materials whose atomic bonding is mainly from electrostatic interactions, on the other hand, both valence charge and atomic spacing can contribute to the strength and modulus, and the Keyes parameter K=e^2^/r^4^ (e-electron charge, r-atomic distance) is the key scaling parameter^94^. Limited metallic materials with the same electronic structure show the atomic spacing to be the only decisive parameter for elastic modulus, for example, alkali metals, some ionic crystals, and oxide crystals. In these crystals, the bulk modulus was observed to scale with interatomic spacing raised to the -4 power^94^. However, when the d-shell electrons become effective in atomic bonding (e.g., in transition metals) or the bonding is more coherent (such as in compounds containing metalloids), the scaling can be complex^94^. In contrast to these crystals, MGs can be envisioned as materials which are randomly densely-packed with isotropic elastic properties, and the elastic constants can be related to each other through equations: 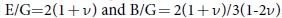, where E, B, 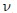 are Young’s modulus, bulk modulus, and Poisson’s ratio, respectively. Consequently, elastic constants of any MG can be derived as a function of λ using the universal correlation (Eq. 2) combined with the isotropic relationship. In other words, λ can be treated as an inherent length parameter conveniently incorporating the influence of both the average atomic distance and effective valence electron charge. It is like the “structure fingerprint” providing the information of elastic properties of MGs.

We want to particularly point out that the universal correlation in MGs (Eqs. 1 and 2) is, in fact, even simpler than that in the elemental metals. Figure 4 is the dependence of shear modulus and bulk modulus on the atomic radii for elemental metals [the original data were taken from refs 103,104]. In contrast to Fig. 2, a more scattered scaling relationship is observed even in metals with the same crystalline structure (for example, in FCC- Cu, Ni, Al, Ag, Au, and Pt, indicated by the asterisks). It is noted that the universal scaling relationship in MGs (Eqs. 1 and 2) is based on the fact that λ actually represents the effect of both atomic spacing and valence electron density. In addition, the correlation between the electronic vector 2k_F_ and the structural vector q_1_, namely, 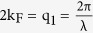 must be valid. This latter correlation is, however, not necessarily true in crystalline metals due to their lattice anisotropy at the atomic level. The atomic bonding directionality as well as interatomic distance in various directions leads to the various slip systems for deformation. A non-spherical Brillouin zone subsequently affects the yield strength and elastic modulus for crystalline metals. Metallic glasses, on the other hand, can be taken as metallic materials having isotropic electron cloud. Furthermore, their atomic structures are lack of long-range periodicity and virtually isotropic (i.e., a better spherical symmetry) even down to the atomic level, leading to the universal scaling relationship observed in Fig. 2. It should be noted that λ is obtained from the statistic structural information of MGs, and there are still many uncertainties and challenges in decoding localized/atomic structure of MGs^105^,^106^. Prolific motifs about structure-properties relationship still merit in-depth investigation^107^. For example, atomic density fluctuation and structural heterogeneity frozen in during rapid quenching is expected to result in the fluctuation of inter-atomic distance and valence electron density around the average value^108^,^109^,^110^, also contributes to the error bar of the universal relationship in Eqs. 1 and 2.

In summary, there exists in metallic glasses a unique characteristic length λ (or q_1_) that exhibits a universal, yet simple, correlation with the mechanical properties such as the modulus and yield strength. The universal relationship appears to be resulted from the fact that metallic glasses are essentially structurally/chemically isotropic as compared to crystalline metals which have certain structural and electronic directionality. Our result demonstrates that, in nature, “more complex” amorphous alloys actually can have a simpler mechanical behavior.

## Additional Information

**How to cite this article**: Wu, Y. *et al.* Inherent structure length in metallic glasses: simplicity behind complexity. *Sci. Rep.* **5**, 12137; doi: 10.1038/srep12137 (2015).

## Figures and Tables

**Figure 1:**
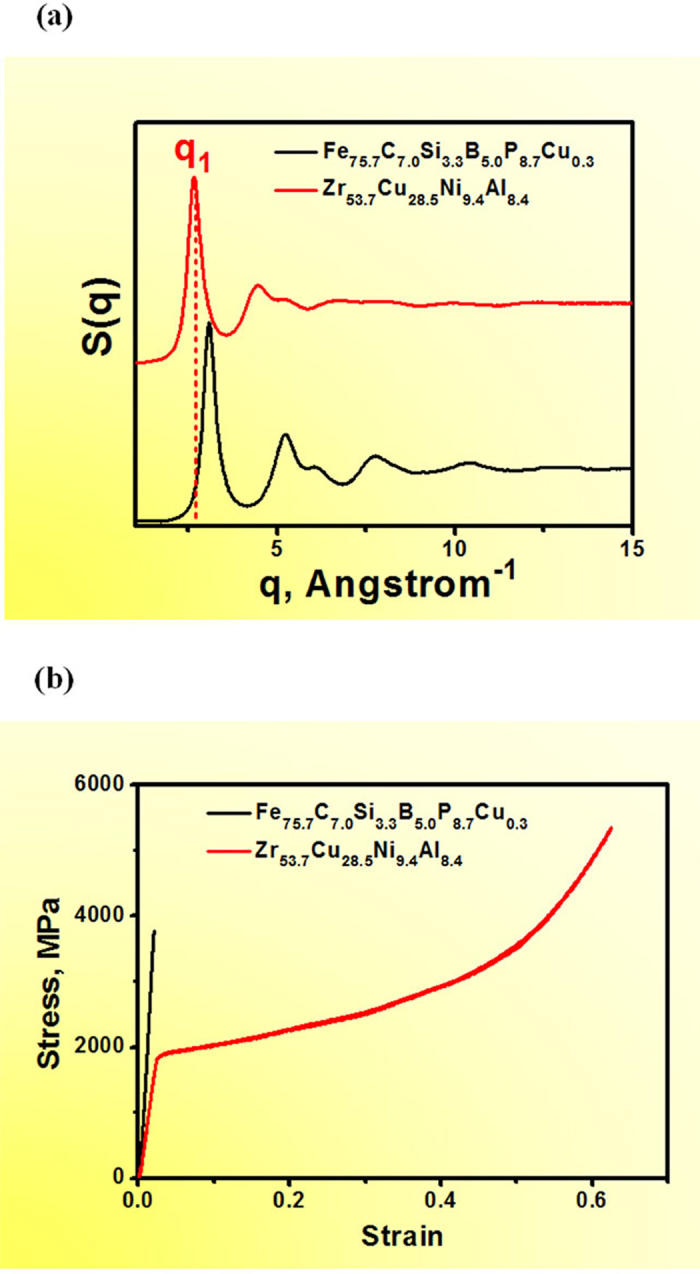
S(q) curves (**a**) and compressive stress-strain curves (**b**) of two representative Fe- and Zr-based BMGs, showing distinct structural characteristics and deformation behavior.

**Figure 2:**
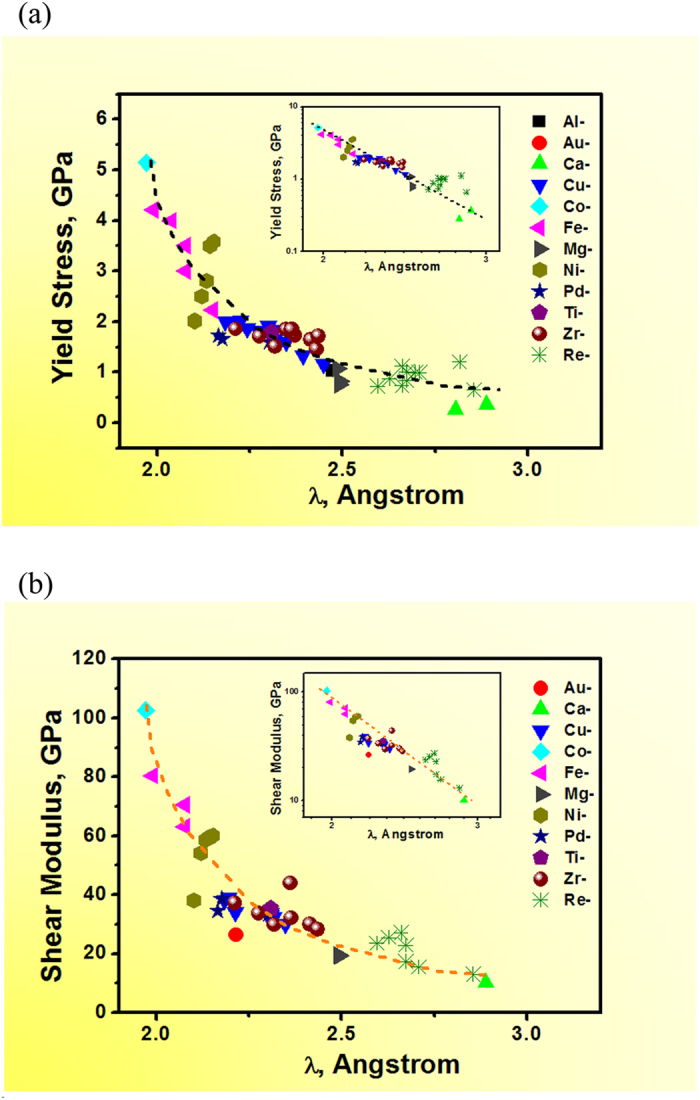
Correlation between yield strength (**a**), shear modulus (**b**), and the characteristic wavelength, λ for different BMGs;A universal relationship can be observed. The inset shows the corresponding log-log plot.

**Figure 3:**
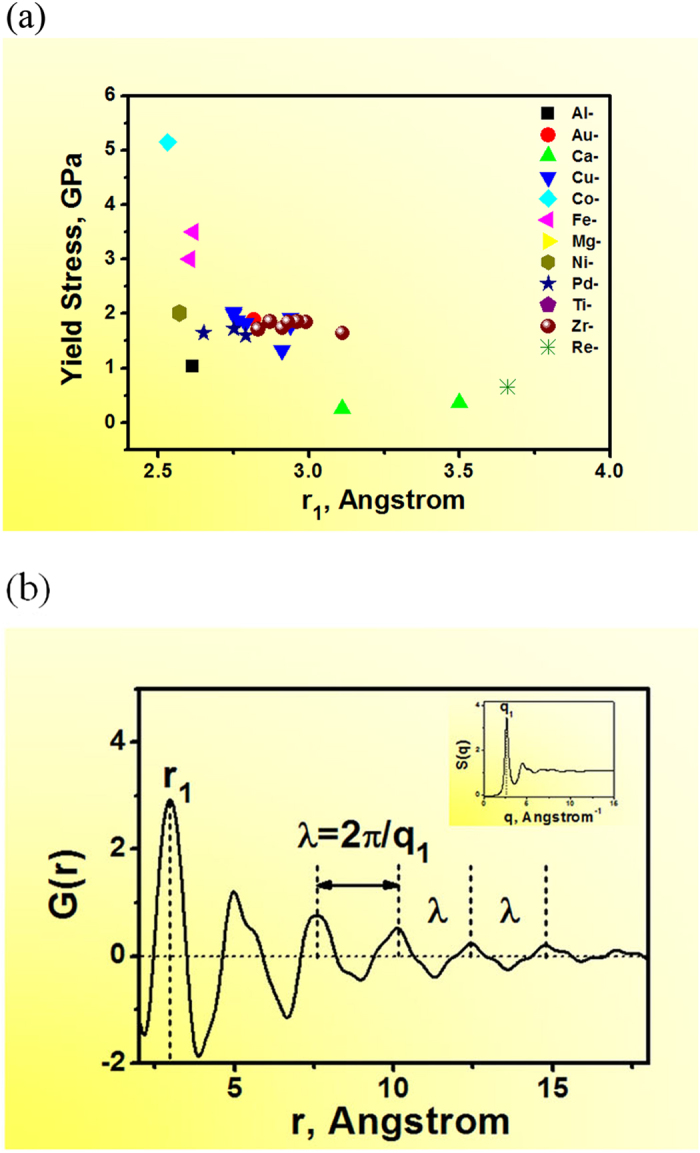
Dependence of the yield strength of MGs on the nearest neighbour distance [r_1_ on g(r)] (**a**); no distinct correlation can be observed. Schematic illustration of the meaning of r_1_ and λ on a typical g(r) curve of Zr-based MG (**b**), and inset is its corresponding S(q) curve.

**Figure 4:**
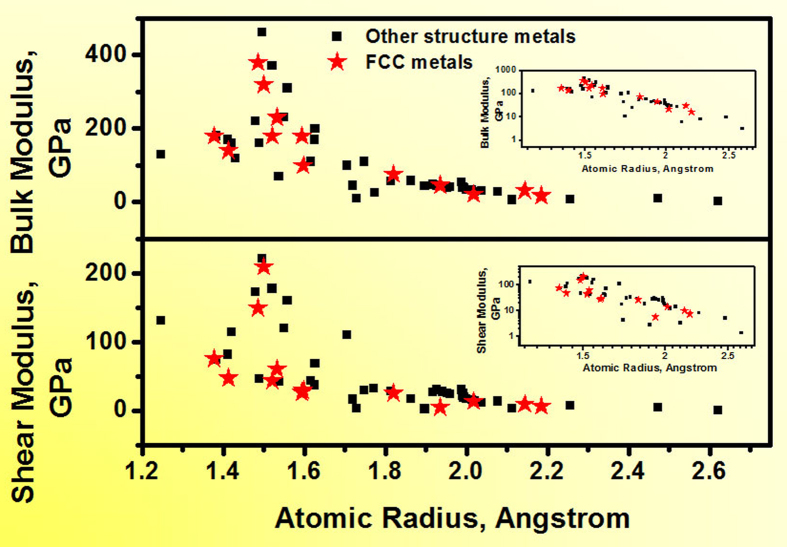
Dependence of the shear and bulk modulus on the atomic radius of most elemental metals. The stars represent the crystalline metals with the FCC structure.

**Table 1:** 

Alloy	q_1_,Å^−1^	r_1_, Å	σ_y_,GPa	B,GPa	G,GPa	E,GPa	Refs.
Al_89_La_6_Ni_5_	2.541	2.612	1.033				25, 26, 27
Au_49_Ag_5.5_Pd_2.3_Cu_26.9_Si_16.3_	2.836	2.816	1.12	132.3	26.5	74.4	28,29
Ca_65_Mg_15_Zn_20_	2.174	3.5	0.364	22.6	10.1	22.4	30,31
Ca_60_Mg_15_Cu_25_	2.239	3.11	0.252				32,33
Cu_65_Zr_35_	2.822	2.75	2.019				34,35
Cu_64_Zr_36_	2.838		1.944	104.3	34	92	34, 35, 36, 37, 38
Cu_61.4_Zr_38.6_	2.801	2.76	1.87				34,35
Cu_56_Zr_44_	2.763	2.79	1.82				34,35,39
Cu_50_Zr_50_	2.719	2.94	1.772	101.2	31.3	85	34,40, 41, 42
Cu_46_Zr_54_	2.674		1.5926	128.5	30	83.5	34,43,44
Cu_40_Zr_60_	2.656	2.91	1.333				45,46
Cu_33.3_Zr_66.7_	2.564		1.17				46‡
Cu_48_Zr_48_Al_4_	2.71		1.65	113.7	32.4	88.7	47, 48, 49‡
Cu_47.5_Zr_47.5_Al_5_	2.72		1.742	113.7	33	87	47, 48, 49‡
Cu_47_Zr_47_Al_6_	2.72		1.834	113.8	33.8	92.4	47,48,50‡
Cu_46_Zr_46_Al_8_	2.73	2.94	1.926	116.4	34.3	93.7	50,51‡
Cu_47_Ti_33_Zr_11_Ni_8_Si_1_	2.875	2.75	2		38.3	100	52,53
Cu_47_Ti_33_Zr_11_Ni_8_Fe_1_	2.86		2.008		39	102	53,54
Co_43_Fe_20_Ta_5.5_B_31.5_	3.186	2.53	5.147	209	102.7	268	55, 56, 57, 58‡
Fe_76.0_C_7.0_Si_3.3_B_5.0_P_8.7_	3.025	2.605	3		63.2	165	59^‡^
Fe_75.7_C_7.0_Si_3.3_B_5.0_P_8.7_Cu_0.3_	3.025	2.615	3.5		70.58	184.2	59^‡^
[(Fe_0.5_Co_0.5_)_0.75_B_0.2_Si_0.05_]_96_Nb_4_	3.156		4.21		80.46	210	60,61
Fe_40_Ni_40_P_14_B_6_	2.922		2.23				62,63
Fe_66_Nb_4_B_30_	3.082		4				64,65
Mg_65_Cu_25_Gd_10_	2.519		0.83	46.3	19.3	50.6	13,66
Mg_65_Cu_25_Tb_10_	2.52		0.76	44.7	19.6	51.3	13,67,68
Mg_61_Cu_28_Gd_11_	2.526		1.075				69,70
Ni_60_Nb_37_Sn_3_	2.944		2.8		58.6	198.6	71
Ni_60_Nb_35_Sn_5_	2.962		2.5	267	54.1	183.2	71
Ni_60_Sn_6_(Nb_0.8_Ta_0.2_)_34_	2.93		3.5	189	59.41	161.3	72,73
Ni_60_Sn_6_(Nb_0.6_Ta_0.4_)_34_	2.917		3.58	197.6	60.1	163.7	72,73
Ni_60_Pd_20_P_20_	2.99	2.57	2.0				74
Ni_60_Pd_20_P_17_B_3_	2.99	2.57	2.022	181	38	106	75,76
Pd_80_Si_20_	2.727	2.79	1.3	182.6	33.4	94.5	77,78
Pd_40_Ni_40_P_20_	2.886	2.65	1.65	184.9	38.6	108	79
Pd_40_Cu_30_Ni_10_P_20_	2.9	2.75	1.72	146	34.5	92	80, 81, 82
Ti_40_Zr_25_Ni_3_Cu_12_Be_20_	2.72		1.8	109.6	35.5	96.2	13‡
Zr_57_Ti_5_Cu_20_Ni_8_Al_10_	2.602	3.11	1.65	99.2	30.1	82	13,83
Zr_55_Cu_35_Al_10_	2.649	2.91	1.74				84
Zr_41_Ti_14_Cu_12.5_Ni_10_Be_22.5_	2.84	2.87	1.86	114.7	37.4	101.3	13,85
Zr_53.7_Cu_28.5_Ni_9.4_Al_8.4_	2.675	2.99	1.85			85	‡
Zr_52.5_Ti_5_Cu_17.9_Ni_14.6_Al_10_	2.658	2.96	1.86	114.1	32.3	88.6	13,45
Zr_64.13_Cu_15.75_Ni_10.12_Al_10_	2.58		1.721	106.63	28.46	78.41	13,86
Zr_62_Al_8_Ni_13_Cu_17_	2.585		1.46				87
Zr_46_Cu_37.6_Ag_8.4_Al_8_	2.76	2.83	1.716	115.5	33.8	92.4	88‡
Zr_53.8_Cu_31.6_Ag_7_Al_7.6_	2.71		1.518	106	29.9	82	88
Zr_48_Cu_36_Al_8_Ag_8_	2.66	2.93	1.85		44	115	89‡
La_62_Al_14_(Cu_5/6_Ag_1/6_)_14_(Ni_1/2_Co_1/2_))_10_	2.2	3.66	0.65	41	13	35	13,90,91
Y_55_Al_25_Co_20_	2.23		1.203				13,45,92
La_55_Al_25_Co_20_	2.32		0.989	39.34	15.42	40.9	13,45,92
Pr_55_Al_25_Co_20_	2.35		1.007	43.48	17.35	45.9	13,45,92
Nd_55_Al_25_Co_20_	2.33		0.996				13,45,92
Gd_55_Al_25_Co_20_	2.36		0.734				13,45,92
Tb_55_Al_25_Co_20_	2.35		0.834	50.19	22.85	59.53	13,45,92
Dy_55_Al_25_Co_20_	2.42		0.717	52.22	23.52	61.36	13,45,92
Ho_55_Al_25_Co_20_	2.39		0.869	58.81	25.42	66.64	13,45,92
Er_55_Al_25_Co_20_	2.36		1.117	60.7	27.08	70.72	13,45,92

Summary of the structural information and mechanical properties of BMGs, including first diffraction peak on the S(q) and G(r) curves, yield strength, and elastic moduli. The experimental procedure can be found in refs 35,59,90.

^‡^data from the present work.

## References

[b1] Courtney T. H. Mechanical Behavior of Materials (McGraw-Hill, 2000).

[b2] Ashby M. F. & Greer A. L. Metallic glasses as structural materials. Scripta Mater 54, 321–326 (2006).

[b3] Cahn R. W. Physical Metallurgy (North-Holland, 1970).

[b4] Feng D. Metal Physics (Science Press, Beijing, 1999).

[b5] Friedel J. Dislocations. (Pergamon Press, New York, 1964).

[b6] Chen M. W. *et al.* Deformation twinning in nanocrystalline aluminum. Science 300, 1275–1277 (2003). 12714676 10.1126/science.1083727

[b7] Ma E. *et al.* Strain hardening and large tensile elongation in ultrahigh-strength nano-twinned copper. Appl. Phys. Lett. 85, 4932 (2004).

[b8] Lu K., Lu L. & Suresh S. Strengthening materials by engineering coherent internal boundaries at the nanoscale. Science 324, 349 (2009). 19372422 10.1126/science.1159610

[b9] Yang B., Liu C. T. & Nieh T. G. Unified equation for the strength of bulk metallic glasses. Appl. Phys. Lett. 88, 221911 (2006).

[b10] Guan P. F., Chen M. W. & Egami T. Stress-Temperature Scaling for Steady-State Flow in Metallic glasses. Phys. Rev. Lett. 104, 205701 (2010). 20867037 10.1103/PhysRevLett.104.205701

[b11] Johnson W. L. & Samwer K. A universal criterion for plastic yielding of metallic glasses with a (T/Tg)2/3 temperature dependence. Phys. Rev. Lett. 95, 195501 (2005). 16383993 10.1103/PhysRevLett.95.195501

[b12] Cheng Y. Q., Sheng H. W. & Ma E. Relationship between structure, dynamics, and mechanical properties in metallic glass-forming alloys. Phys. Rev. B 78, 014207 (2008).

[b13] Wang W. H. The elastic properties, elastic models and elastic perspectives of metallic glasses. Prog. Mater. Sci. 57, 487–656 (2012).

[b14] Ma D., Stoica A. D. & Wang X. L. Power-law scaling and fractal nature of medium-range order in metallic glasses. Nat. Mater. 8, 30–34 (2009). 19060888 10.1038/nmat2340

[b15] Elliott S. R. Medium-range Structural Order in Covalent Amorphous Solids. Nature 345, 445–452 (1991).

[b16] Gottlicher J. & Pentinghaus H. J. Compositional influence on shape and position of the first sharp diffraction peak (FSDP) in silicate and germinate glasses. Ber. Bunsenges. Phys. Chem. 100, 1563–1568 (1996).

[b17] Rouxel T. Elastic properties and short-to medium-range order in glasses. J. Am. Ceram. Soc. 90, 3019–3039 (2007).

[b18] Haussler P. Interrelations between atomic and electronic structures-Liquid and amorphous metals as model systems. Phys. Rep. 222, 65–143 (1992).

[b19] Egami T.& Billinge S. J. L. Underneath the bragg peaks—structural analysis of complex materials. (Pergamon) (2003).

[b20] Du J. C. & Corrales L. R. Compositional dependence of the first sharp diffraction peaks in alkali silicate glasses: A molecular dynamics study. J. Non-Cryst. Sol. 352, 3255–3269 (2006).

[b21] Massalski T. B. & Mizutani U. Electronic structure of Hume-rothery phases. Prog. Mater. Sci. 22, 151–262 (1978).

[b22] Hessinger J., White B. E. & Pohl R. O. Elastic properties of amorphous and crystalline ice films. Planet. Space Sci. 44, 937–944 (1996).

[b23] Varshneya A. K. Fundamentals of inorganic glasses. (Academic Press Inc., Boston, 1994).

[b24] Bridge B., Patel N. D. & Waters D. N. On the elastic constants and structure of the pure inorganic oxide glasses. Phys. Stat. Sol. (a) 77, 655–668 (1983).

[b25] Sheng H. W. *et al.* Atomic packing in multicomponent aluminum-based metallic glasses. Acta Mater. 56, 6264–6272 (2008).

[b26] Li G. Q. *et al.* Local structure variations in Al_89_La_6_Ni_5_ metallic glass. Acta Mater. 57, 804–811 (2009).

[b27] Guo M. L. *et al.* Thermal Stability and Mechanical Properties of Spray-Formed and Melt-Spun Al_89_La_6_Ni_5_ Metallic glass Matrix Composites, Materials Transactions JIM 48, 1717–1721 (2007).

[b28] Wang X. D. *et al.* Atomic-level structural modifications induced by severe plastic shear deformation in bulk metallic glasses Scripta Mater. 64, 81–84 (2011).

[b29] Wang L., Lu Z. P. & Nieh T. G. Onset of yielding and shear band nucleation in an Au-based bulk metallic glass. Scripta Mater. 65, 759–762 (2011).

[b30] Senkov O. N. & Scott J. M. Glass forming ability and thermal stability of ternal Ca-Mg-Zn bulk metallic glasses. J. Non-crystalline Solids 351, 3087–3094 (2005).

[b31] Wang G. Y., Liaw P. K., Senkov O. N. & Miracle D. B. The duality of fracture behavior in a Ca-based bulk metallic glass. Metallurgical and materials transactions A 42, 1499–1503 (2011).

[b32] Barney E. R. *et al.* A neutron and X-ray diffraction study of Ca-Mg-Cu metallic glasses. Intermetallics 19, 860–870 (2011).

[b33] Senkov O. N., Scott J. M. & Miracle D. B. Composition range and glass forming ability of ternary Ca-Mg-Cu bulk metallic glasses. J. Alloys Compnds. 424, 394–399 (2006).

[b34] Calvayrac Y. *et al.* On the stability and structure of Cu-Zr based glasses. Phi. Mag. B 48, 323–332 (1983).

[b35] Liu X. J. *et al.* Metallic liquids and glasses: atomic order and global packing. Phys. Rev. Lett. 105, 155501 (2010). 21230918 10.1103/PhysRevLett.105.155501

[b36] Kwon O. J. *et al.* Thermal and mechanical behaviors of Cu-Zr amorphous alloys. Mater. Sci. Eng. A 449, 169–171 (2007).

[b37] Mattern N. *et al.* Short-range order of Cu-Zr metallic glasses. J. Alloys Compnds. 485, 163–169 (2009).

[b38] Ott R. T. *et al.* Strain dependence of peak widths of reciprocal- and real-space distribution functions of metallic glasses from *in situ* x-ray scattering and molecular dynamics simulations. Phys. Rev. B 80, 064101 (2009).

[b39] Park K. W. *et al.* Atomic packing density and tis influence on the properties of Cu-Zr amorphous alloys. Scripta Mater. 57, 805–808 (2007).

[b40] Eckert J. *et al.* High strength ductile Cu-base metallic glass. Intermetallics 14, 876–881 (2006).

[b41] Baser T. A., Das J., Eckert J. & Baricco M. Glass formation and mechanical properties of (Cu_50_Zr_50_)_100-x_Al_x_(x=0,4,5,7) bulk metallic glasses. J. Alloys and Compnds. 483, 146–149 (2009).

[b42] Mattern N. *et al.* Structural evolution of Cu-Zr metallic glasses under tension. Acta Mater. 57, 4133–4139 (2009).

[b43] Kwon O. J. *et al.* Thermal and mechanical behaviors of Cu-Zr amorphous alloys. Mater. Sci. Eng. A 449, 169–171 (2007).

[b44] Xu D. H., Duan G. & Johnson W. L. Unusual glass-forming ability of bulk amorphous alloys based on ordinary metal copper. Phys. Rev. Lett. 92, 245504 (2004). 15245096 10.1103/PhysRevLett.92.245504

[b45] Ma D., Stoica A. D. & Wang X. L. Power-law scaling and fractal nature of medium-range order in metallic glasses. Nat. Mater. 8, 30–34 (2009). 19060888 10.1038/nmat2340

[b46] Lu B. F., Li J. F. L., Kong T. & Zhou Y. H. Correlation between mechanical behavior and glass forming ability of Zr-Cu metallic glasses. Intermetallics 19, 1032–1035 (2011).

[b47] Eckert J. *et al.* High strength ductile Cu-base metallic glass. Intermetallics 14, 876–881 (2006).

[b48] Das J. *et al.* “Work-hardenable” ductile bulk metallic glass. Phys. Rev. Lett. 94, 205501 (2005). 16090260 10.1103/PhysRevLett.94.205501

[b49] Baser T. A., Das J., Eckert J. & Baricco M. Glass formation and mechanical properties of (Cu_50_Zr_50_)_100-x_Al_x_(x=0,4,5,7) bulk metallic glasses. J. Alloys and Compnds. 483, 146–149 (2009).

[b50] Yu P., Bai H. Y., Tang M. B. & Wang W. H. Excellent glass-forming ability in simple Cu_50_Zr_50_-based alloys. J. Non-Crystalline Solids 351, 1328–1332 (2005).

[b51] Wang X. D. *et al.* Atomic structure and glass forming ability of Cu_46_Zr_46_Al_8_ bulk metallic glass. J. Appl. Phys. 104, 093519 (2008).

[b52] Bednarcik J. *et al.* Microstructural changes induced by thermal treatment in Cu_47_Ti_33_Zr_11_Ni_8_Si_1_ metallic glass. Mater. Sci. Eng. A 498, 335–340 (2008).

[b53] Calin M. *et al.* Glass formation and crystallization of Cu_47_Ti_33_Zr_11_Ni_8_×_1_(X=Fe, Si, Sn, Pb) alloys. Mater. Sci. Eng. A 392, 169–178 (2005).

[b54] Calin M., Eckert J. & Schultz L. Improved mechanical behavior of Cu-Ti-based bulk metallic glass by *in situ* formation of nanoscale precipitates. Scripta Mater. 48, 653–658 (2003).

[b55] Kaban I. *et al.* Topological and chemical ordering in Co_43_Fe_20_Ta_5.5_B_31.5_ metallic glass. Phys. Rev. B 79, 212201 (2009).

[b56] Inoue A. *et al.* Cobalt-based bulk glassy alloy with ultrahigh strength and soft magnetic properties. Nature Mater. 2, 661–663 (2003). 14502274 10.1038/nmat982

[b57] Hostert C. *et al.* Ab initio molecular dynamics model for density, elastic properties and short range order of Co-Fe-Ta-B metallic glass thin films. J. Phys.: Condens. Matter 23, 475401 (2011). 22056956 10.1088/0953-8984/23/47/475401

[b58] Wang W. H. Correlations between elastic moduli and properties in bulk metallic glasses. J. Appl. Phys. 99, 093506 (2006).

[b59] Li H. X. *et al.* Correlation of nano-clustering and glass formation in alloys with a high solvent content. Scientific Report 3, 1983 (2013). 10.1038/srep01983 PMC3680804 23760427

[b60] Stoica M. *et al.* Thermal stability and magnetic properties of FeCoBSiNb bulk metallic glasses. J. alloys Compnds. 504, S123–S128 (2010).

[b61] Shen B. L., Inoue A. & Chang C. T. Superehigh strength and good soft-magnetic properties of (Fe,Co)-B-Si-Nb bulk glassy alloys with high glass-forming ability. Appl. Phys. Lett. 85, 4911 (2004).

[b62] Petkov V. Energy-dispersive X-ray diffraction analysis of the structure of disordered materials. J. Non-Crystalline Solids 192, 65–68 (1995).

[b63] Yao K. F. & Zhang C. Q. Fe-based bulk metallic glass with high plasticity. Appl. Phys. Lett. 90, 061901 (2007).

[b64] Stoica M. *et al.* Crystallization kinetics and magnetic properties of Fe_66_Nb_4_B_30_ bulk metallic glass. J. Alloys Compnds. 483, 632–637 (2009).

[b65] Stoica M., Hajlaoui K., Lemoulec A. & Yavari A. R. New ternary Fe-based bulk metallic glass with high boron content. Phil. Mag. Lett. 86, 267–275 (2006).

[b66] Park E. S. *et al.* Correlation between plasticity and fragility in Mg-based bulk metallic glasses with modulated heterogeneity. J. Appl. Phys. 104, 023520 (2008).

[b67] Zhang Q. R. *et al.* Effect of substitution of Ni for Cu on glass-forming ability and mechanical properties of Mg-Cu-Tb metallic glass alloys. The Chinese Journal of Nonferrous Metals 17, 303–307 (2007).

[b68] Xi X. K., Zhao D. Q., Pan M. X. & Wang W. H. Highly processable Mg_65_Cu_25_Tb_10_ bulk metallic glass. J. Non-Crystalline Solids 344, 189–192 (2004).

[b69] Wang X. D. *et al.* Atomic packing in Mg_61_Cu_28_Gd_11_ bulk metallic glass. Appl. Phys. Lett. 98, 031901 (2011).

[b70] Zheng Q. *et al.* Critical size and strength of the best bulk metallic glass former in the Mg-Cu-Gd ternary system. Scripta Mater. 56, 161–164 (2007).

[b71] Yim H. C. *et al.* Structure and mechanical properties of bulk glass-forming Ni-Nb-Sn alloys. Scripta Mater. 54, 187–190 (2006).

[b72] Schuh C. A. & Nieh T. G. A survey of instrumented indentation studies on metallic glasses. J. Mater. Res. 19, 46–57 (2004).

[b73] Yim H. C., Tokarz M., Bilello J. C. & Johnson W. L. Structure and properties of Ni_60_(Nb_100-x_Ta_x_)_34_Sn_6_ bulk metallic glass alloys. J. Non-crystalline Solids 352, 747–755 (2006).

[b74] Zeng Y. Q., Inoue A., Nishiyama N. & Chen M. W. Ni-rich Ni-Pd-P bulk metallic glasses with significantly improved glass-forming ability and mechanical propertis by Si addition. Intermetallics 18, 1790–1793 (2010).

[b75] Kawashima A. *et al.* Mechanical properties of a Ni_60_Pd_20_P_17_B_3_ bulk glassy alloy at cryogenic temperatures. Mater. Sci. Eng. A 498, 475–481 (2008).

[b76] Matsuura M. *et al.* Local atomic structure of Ni_60_Pd_20_P_20_ and Ni_60_Pd_20_P_17_B_3_ bulk metallic glasses and the origin of glass forming ability. J. Alloys Compnds. 496 135–139 (2010).

[b77] Petkov V. Energy-dispersive X-ray diffraction analysis of the structure of disordered materials. J. Non-Crystalline Solids 192, 65–68 (1995).

[b78] Yao K. F., Ruan F., Yang Y. Q. & Chen N. Superductile bulk metallic glass. Appl. Phys. Lett. 88, 122106 (2006).

[b79] Chen N. *et al.* Influence of minor Si addition on the glass-forming ability and mechanical properties of Pd_40_Ni_40_P_20_ alloy. Acta Mater. 57, 2775–2780 (2009).

[b80] Mattern N. *et al.* Structural behavior and glass transition of bulk metallic glasses. J. Non-Crystalline Solids 345, 758–761 (2004).

[b81] Hirata A., Hirotsu Y., Kuboya S. & Nieh T. G. Local structural fluctuation in Pd-Ni-P bulk metallic glasses examined using nanobeam electron diffraction. J. Alloys Compnds. 483, 64–69 (2009).

[b82] Haruyama O., Sugiyama K. Sakurai M. & Waseda Y. A local structure change of bulk Pd_40_Ni_40_P_20_ glass during full relaxation. J. Non-Crystalline Solids 353, 3053–3056 (2007).

[b83] Hufnagel T. C., Ei-Deiry P. & Vinci R. P. Development of shear band structure during deformation of a Zr_57_Ti_5_Cu_20_Ni_8_Al_10_ bulk metallic glass. Scripta Mater. 43, 1071–1075 (2000).

[b84] Fan C. *et al.* Pair distribution function study and mechanical behavior of as-cast and structurally relaxed Zr-based bulk metallic glasses. Appl. Phys. Lett. 89, 231920 (2006).

[b85] Hui X. *et al.* Atomic structure of Zr_41.2_Ti_13.8_Cu_12.5_Ni_10_Be_22.5_ bulk metallic glass alloy. Acta Mater. 57, 376–391 (2009).

[b86] Liu Y. H. *et al.* Super plastic bulk metallic glasses at room temperature. Science 315, 1385–1388 (2007). 17347434 10.1126/science.1136726

[b87] Wang X. D. *et al.* Tensile behavior of bulk metallic glasses by *in situ* x-ray diffraction. Appl. Phys. Lett. 91, 081913 (2007).

[b88] Wang X. *et al.* A plastic Zr-Cu-Ag-Al bulk metallic glass. Acta Mater. 59, 1037–104 (2011).

[b89] Zhang Q. S., Zhang W. & Inoue A. New Cu-Zr-based bulk metallic glasses with large diameters of up to 1.5cm, Scripta Mater. 55, 711–713 (2006).

[b90] Bednarcik J. *et al.* Thermal expansion of La-based BMG studied by *in situ* high-energy X-ray diffraction. J. Alloys Compnds. 504S, S155–S158 (2010).

[b91] Jiang Q. K. *et al.* La-based bulk metallic glasses with critical diameter up to 30 mm. Acta Mater. 55, 4409 (2007).

[b92] Li S. *et al.* Formation and properties of RE_55_Al_25_Co_20_(RE=Y, Ce, La, Pr, Nd, Gd, Tb, Dy, Ho and Er) bulk metallic glasses. J. Non-Crystalline Solids 354, 1080–1088 (2008).

[b93] Gilman J. J. Electronic basis of the strength of materials. (Cambridge University Press 2003).

[b94] Gilman J. J. Micromechanics of Flow in Solids. (McGraw-Hill 1969).

[b95] Hirata A. *et al.* Direct observation of local atomic order in a metallic glass. Nature Mater. 10, 28–33 (2011). 21102454 10.1038/nmat2897

[b96] Mizutani U. Electronic structure of metallic glasses. Prog. Mater. Sci. 28, 97–228 (1983).

[b97] Hafner J. & von Heimendahl L. Microscopic calculations of the stability of metallic glasses. Phys. Rev. Lett. 42, 386–389 (1979).

[b98] Nagel S. R. & Tauc J. Nearly-free-electron approach to the theory of metallic glass alloys. Phys. Rev. Lett. 35, 380–383 (1975).

[b99] Han G. *et al.* The e/a values of ideal metallic glasses in relation to cluster formulae. Acta Mater. 59, 5917–5923 (2000).

[b100] Yu H. B., Wang W. H. & Bai H. Y. An electronic structure perspective on glass-forming ability in metallic glasses. Appl. Phys. Lett. 96, 081902 (2010).

[b101] Rose J. H., Ferrante J. & Smith J. R. Universal binding energy curves for metals and bimetallic interfaces. Phys. Rev. Lett. 47, 675 (1981).

[b102] Banerjea A. & Smith J. R. Origins of the universal binding-energy relation. Phys. Rev. B 37, 6632 (1988). 10.1103/physrevb.37.6632 9943929

[b103] King H. W. & Pettifor D. G. in Physical Metallurgy , 3rd ed. Edited by Cahn R. W. & Hassen P. (North-Holland, Amsterdam 1983).

[b104] Ashcroft N. W. & Mermin N. D. Solid state physics. (Harcourt, inc. 1976).

[b105] Lee C. Y., Stachurski Z. H. & Welberry T. R. The geometry, topology and structure of amorphous solids. Acta Mater. 58, 615–625 (2010).

[b106] Wu Z. W., Li M. Z., Wang W. H. & Liu K. X. Hidden topological order and its correlation with glass-forming ability in metallic glasses. Nat. Comm. 6, 6035 (2014). 10.1038/ncomms7035 25580857

[b107] Cheng Y. Q. & Ma E. Atomic-level structure and structure-property relationship in metallic glasses. Prog. Mater. Sci. 56, 379–473 (2011).

[b108] Liu Y. H. *et al.* Characterization of Nanoscale Mechanical Heterogeneity in a Metallic Glass by Dynamic Force Microscopy. Phys. Rev. Lett. 106, 125504 (2011). 21517325 10.1103/PhysRevLett.106.125504

[b109] Wisitsorasak A. & Wolynes P. G. On the strength of glasses. PNAS 1009, 16068 (2012). 22988070 10.1073/pnas.1214130109 PMC3479550

[b110] Wagner H. *et al.* Local elastic properties of a metallic glass. Nat. Mater. 10, 439–442 (2011). 21602807 10.1038/nmat3024

